# Supplementary material for: Mapping the genealogy of medical device predicates in the United States
Source: PLoS One. 2021 Oct 7;16(10):e0258153. doi: 10.1371/journal.pone.0258153 (PMC8496833; doi:10.1371/journal.pone.0258153)
Supplement: Attachment — Submitted filename: Response to Reviewers.docx [file pone.0258153.s001.docx]

**PLOS One**

**Manuscript:** “Mapping the genealogy of medical device predicates in the Unites States”
Response to Reviewers

I would like to thank the reviewers (Dr. Borissov and Reviewer#1) and the academic editor (Dr. Mordaunt) for their very helpful comments and constructive criticism about my manuscript. I have attempted to address the various concerns raised in the review, and believe that these changes have allowed me to considerably strengthen the manuscript.

**Reviewer #1:** *the use of predicate / child and predicate / children terms is confusing. suggest chage to antecedent / sucessor or similar.*

**Author Response:** I agree that the terminology can be confusing. Since the predicate term is a defined term in regulatory use, and is used by publications that have been tracking medical device safety and effectiveness issues (such as the references cited by the reviewers), I provided a definition of the “predicate” word to stay true to that usage. Additionally, instead of using the “children” term, I steered clear of that language; instead using ancestral tree of the predicate (this is how other publications referred to by the reviewers define them), and brought in the word “descendant” to describe the devices that come from the predicate’s ancestral tree. I minimized the use of the predicate-child relation language as much as possible; I do hope this makes the terminology clearer.

Reviewer #1: *The discussion section includes statements that are not supported with data that is obvious eg "prodiuct codes with more class 2 recalls tended to have more devices and more complaints" is not supported by data and analysiss within the body of the paper.*

**Author Response:** I thank the reviewer for pointing this out. I have added text in the discussion to provide more clarity about Figure 3, and how the size of the data points visualized is proportional to the number of different devices in a particular product code. I hope that the added/amended text in the discussion section clarifies this point.

Reviewer #1: *similarly the conlcusions are unsupported by data or anlsyis from the body of the paper - for example the statement that ".. the technological characteristics of the predicate and its child can fluctuate wiedly, suggetsing a wide vaiation in the accepted characteristics of the substantial equivalence test." both the absence of supprting data and the volubl language will need to be adressed.*

**Author Response:** I agree with the reviewer that the statement in the conclusion that the reviewer points out is not supported by data in the manuscript. The corresponding text in the conclusion has been deleted.

**Reviewer #2:** *Major – The study lacks general statement about its limitations. However, the use of bag-of-words model as one of the most popular representation methods for object categorization is associated with a number of uncertainties. The key idea is to quantize each extracted key point into one of visual words, and then represent each image by a histogram of the visual words. For this purpose, a clustering algorithm (e.g., K-means), is generally used for generating the visual words. As K-means tends to ‘starve’ medium density regions in feature space, measures to minimize the loss of information should be implemented and justified. It has been previously demonstrated that the pairwise similarity cannot be computed directly and to show the statistical consistency, Kernel Density Function Estimation / also referred as “Quantization via Kernel Estimation”, or QKE for short/ is encouraged in previous similar researches as an alternative to empirical distribution as “Quantization via Empirical Estimation”, or QEE. Therefore, additional justification is needed how author avoids in the model the problem of ‘code word uncertainty’ and ‘code word plausibility’.*

**Author Response:** I thank the reviewer for indicating that the study lacks general statements about its limitations. I have added a section in the discussion portion indicating the limitations in the current study, as well as the Word2Vec model that I used for this work. I am unsure about the latter point raised by the reviewer, regarding visual words. I believe visual words are used for extracting information content from images (e.g. “cat”, “dog”, from an image containing a cat and/or a dog), and the QKE and QEE approaches apply in that context. However, the current project does not use visual words and does not extract image information in that manner, and hence the visual word issues like ‘code word uncertainty’ and ‘code word plausibility’ would not apply to the current work. I do apologize if this did not come across clearly in my manuscript, and I have added more content in the Materials and Methods section providing details about the Word2Vec model that I used for this project. I hope that this clarifies the method used to develop the mapping.

**Reviewer #2:** *the references are very limited and only 8 /15 in total/ are research articles. The 510(k) ancestry as well as the related safety and effectiveness concerns have been elaborated in a number of rigorously peer- review publications,*< a number of publications are provided, but not shown here for the sake of brevity>

**Author’s Response:** I thank the reviewer for pointing this significant weakness in the manuscript, and appreciate the references that are provided. These have now been added in the appropriate sections of the background content of the manuscript, and I believe have strengthened the quality of the manuscript considerably.

**Reviewer #2:** *more robust explanations are needed to explain the impact of poor quality of PDF files /line 167 onwards/ and the limitations of the OCR /lines 168-171/ to verify the efficacy of the proposed framework by applying it to object recognition.*

**Author’s Response:** Explanation is provided in the discussion section for why OCR use for text extraction yielded significant errors for PDF documents from before 2002.

**Reviewer #2:**  *In testing recalls and complaints for predicate device /lines 176- 199/ - may author elaborate on how many complaints have been found causal.*

**Author’s Response:** The suggestion from the reviewer is an excellent one; however, the TPLC database provided by the FDA does not have temporal resolution within the complaints to allow the establishment of causal relation between the product recalls and the complaints. I have made a mention of this point in the discussion section of the manuscript.
